# Evaluation and Validation of the Modified Reflux Symptom Questionnaire–Electronic Diary in Patients With Persistent Gastroesophageal Reflux Disease

**DOI:** 10.14309/ctg.0000000000000117

**Published:** 2020-01-20

**Authors:** David A. Andrae, Jennifer Hanlon, Mary Lynn Cala, Kayla Scippa, Christina Graham, Brooke Witherspoon, James Z. Shao, David Reasner

**Affiliations:** 1 Endpoint Outcomes, Boston, Massachusetts, USA;; 2 Ironwood Pharmaceuticals, Inc., Boston, Massachusetts, USA;; 3 Endpoint Outcomes, Long Beach, California, USA;; 4 Formerly of Ironwood Pharmaceuticals, Inc., Cambridge, Massachusetts, USA.

## Abstract

**OBJECTIVES:**

This study aimed to examine the validity of the modified Reflux Symptom Questionnaire–electronic Diary (mRESQ-eD) through patient input and psychometric testing of the questionnaire to support use in clinical trials in patients with persistent gastroesophageal reflux disease (GERD) and in accordance with Food and Drug Administration guidance on patient-reported outcome instruments.

**METHODS:**

Cognitive interviews were conducted with patients (n = 30) to evaluate the interpretability and content validity of draft mRESQ-eD items. Patient data from a phase 2b clinical study (ClinicalTrials.gov identifier: NCT02637557) on persistent GERD served to aid in the construction of weekly scores for heartburn severity, regurgitation severity, and total GERD severity. These scores' psychometric properties were also evaluated.

**RESULTS:**

Minor modifications were made to the draft mRESQ-eD based on patient feedback to improve interpretability and clarity of the instrument. Psychometric analysis suggested that an 8-item version of the mRESQ-eD was best suited to the clinical data. The internal consistency was found to be high (Coefficient ω = 0.95). Retest reliability and convergent validity were strong for a heartburn weekly severity score, regurgitation weekly severity score, and total GERD severity score.

**DISCUSSION:**

The final 8-item mRESQ-eD is a reliable and valid instrument with good psychometric properties for use in clinical trials in patients with persistent GERD. The mRESQ-eD may be considered for inclusion in clinical trials for persistent GERD and potentially positioned, in consultation with Food and Drug Administration, as endpoints to characterize treatment benefit.

## INTRODUCTION

Gastroesophageal reflux disease (GERD) is one of the most commonly diagnosed chronic gastrointestinal illnesses (1,2). The symptoms of GERD, most notably heartburn and regurgitation, represent a significant burden on patients' health-related quality of life (3). Patients with persistent GERD—those who experience frequent and bothersome heartburn and regurgitation despite standard proton pump inhibitor (PPI) treatment—represent a sizeable portion of all patients with GERD (i.e., 20.0%–30.0%) (2,4,5). Patients with persistent GERD experience reduced physical and mental health as compared to patients with PPI-responsive GERD (6).

The diagnosis of GERD has been previously based on objective tests and clinician assessments (e.g., pH monitoring, impendence monitoring) or by mucosal injury (e.g., endoscopy); however, there has been a shift to diagnosing GERD based on patient-reported symptoms in conjunction with other previously validated objective assessments (2,7). In addition to including patient-reported symptoms in GERD diagnosis assessments, regulatory authorities have advocated patient-reported outcome (PRO) instruments for measuring treatment benefit and substantiating labeling claims in general and in GERD specifically (8,9). The United States Food and Drug Administration (FDA) Final Guidance for PRO instruments states that instruments should be developed in the intended population of use, be content valid (i.e., contain all necessary concepts related to the condition), and be developed with sufficient patient input (8). The draft FDA Pediatric GERD Guidance details recommendations by the Agency for establishing efficacy requirements among different age cohorts. The Agency recommends measuring GERD signs/symptoms using PRO instruments and has supported adult studies that evaluate reductions in GERD symptoms as the primary endpoint (9).

A targeted literature review, initially conducted in 2014 and updated in 2018, was performed to identify existing PRO instruments that assess the signs and symptoms of GERD. The most promising instrument identified from the 2014 search, the Reflux Symptom Questionnaire–electronic Diary (RESQ-eD), was developed specifically for persistent GERD and in accordance with the FDA Final PRO Guidance (9). Specifically, RESQ-eD development activities included concept elicitation interviews and focus groups with patients diagnosed with persistent GERD, concept selection based on a literature review and patient and expert input, and confirmatory content validity interviews conducted with patients with persistent GERD that participated in a PRO validation study (ClinicalTrials.gov identifier: NCT00703534) (10). Despite the efforts made in developing the RESQ-eD, additional modifications were needed to improve the content validity of the measure with a focus on examining the conceptual overlap of similar concepts and concepts related to regurgitation. The current study reports and discusses the evaluation of the content validity, scoring, and psychometric properties of the modified RESQ-eD (mRESQ-eD) in the persistent GERD population through cognitive interviews and psychometric evaluation based on data from the phase 2b study (ClinicalTrials.gov identifier: NCT02637557).

## METHODS

### Phase 2b study description

**Figure 1.**
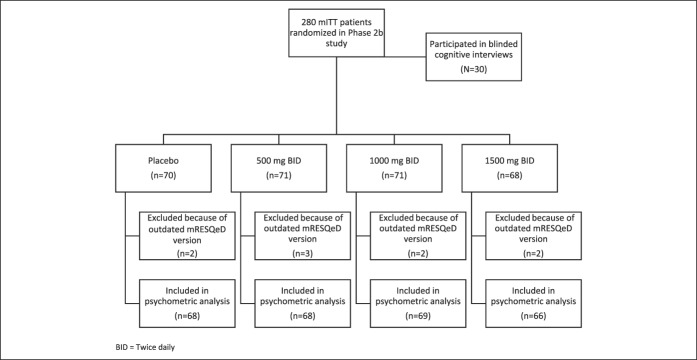
Schematic of patients from phase 2b clinical study who participated in cognitive interviews and whose data were used for psychometric validation purposes. b.i.d. = twice daily; mITT, modified intent to treat; mRESQ-eD, and modified Reflux Symptom Questionnaire–electronic Diary.

The phase 2b study (ClinicalTrials.gov identifier: NCT02637557) was a randomized, double-blinded, placebo-controlled, parallel-group, 8-week study, which aimed to evaluate the safety, efficacy, and dose-response relationship of a bile acid sequestrant in combination with PPI treatment to relieve the symptoms of persistent GERD (11). Experimental treatment arms included 500 mg, 1,000 mg, and 1,500 mg of treatment twice daily. See Figure 1 for a schematic of patient enrollment for cognitive interviews and data use in *post hoc* psychometric analyses.

### RESQ-eD background

The 13-item RESQ-eD was modified based on feedback from FDA reviewers and results of concept elicitation interviews with select patients (n = 30) who participated in a phase 2a study (ClinicalTrials.gov identifier: NCT02030925) for persistent GERD; this led to the development of the 10-item mRESQ-eD, which was debriefed with select participants from the phase 2b study (ClinicalTrials.gov identifier: NCT02637557) (Table 2 for full mRESQ-eD modification history). After these concept elicitation interviews, a preliminary hypothesized conceptual framework was developed containing 3 hypothesized domains (i.e., heartburn, regurgitation/reflux, and other GERD signs/symptoms), which guided instrument development. During the subsequent psychometric analysis, coughing was assessed using 2 different dimensions (i.e., severity and frequency) in the 10-item mRESQ-eD to further explore which dimension best assessed the symptom.

### Exploration of content validity: cognitive interviews

The content validity of the 10-item mRESQ-eD was evaluated in cognitive interviews with select patients from the phase 2b study conducted between March 2016 and May 2017. A convenience subset of patients (i.e., 30) was included in the cognitive interviews; this sample size exceeds the minimum number of patients recommended for cognitive interviews (12). The goal of the cognitive interviews was to determine whether all included items measured distinct and relevant concepts and were supported by patient input. The cognitive interviews were included as an amendment to the study protocol, which was submitted by the sponsor for review and approval by Schulman Independent Review Board (approval number: 201505591, August 12, 2016). During the interviews, patients completed the 10-item mRESQ-eD using a “think-aloud” method to verbalize their thoughts while completing the instrument (13). The interviews consisted of targeted questions as included in an interviewer's guide to obtain patient perception of how well the mRESQ-eD captured their overall experience of symptoms related to GERD. Specifically, the interviews explored each patient's interpretation of the instrument, including whether the patient understood the concepts as intended, ease of completion, clarity, appropriateness of format, response scales, and recall period. All audio recordings were transcribed verbatim, anonymized, and coded using processes guided by established qualitative research methods, including grounded theory and constant comparison method (14,15). The coding scheme was applied using ATLAS.ti version 7.5.17 or higher (Atlas.ti GmbH, Berlin).

### Psychometric evaluation of the mRESQ-eD

**Figure 2.**
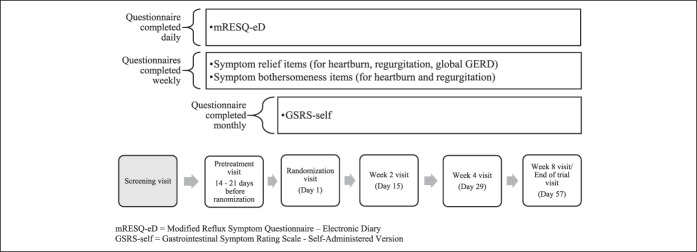
Questionnaire administration schedule during phase 2b study for use in psychometric validation analyses from pretreatment to end of study. GERD, gastroesophageal reflux disease; GSRS-Self, Gastrointestinal Symptom Rating Scale–Self-Administered Version; mRESQ-eD, and modified Reflux Symptom Questionnaire—electronic Diary.

After cognitive interviews, the mRESQ-eD underwent psychometric testing to assure integrity of the instrument scoring and also to define relevant subdomains. The psychometric properties of the mRESQ-eD were confirmed using data from the phase 2b study modified intent to treat the population. Data from 271 patients were included for analysis. In addition to the mRESQ-eD items, several other outcome measures were collected and used for psychometric evaluation. Symptom bothersomeness was assessed by two 7-day recall items, one each for heartburn and regurgitation. Both items were assessed on a 5-point ordinal scale. Responses to the items ranged from 1 (not at all) to 5 (an extreme amount). Symptom relief was measured by one heartburn item, one regurgitation item, and a single global GERD item using a 7-point Likert-type scale with responses ranging from 1 (significantly relieved) to 4 (no change) to 7 (significantly worse). These items were administered weekly throughout the clinical study. In addition, the Gastrointestinal Symptom Rating Scale–Self-Administered Version (GSRS-Self) was completed by patients at randomization, after 4 weeks of treatment, and at the end of treatment (16). The GSRS-Self is a 15-item questionnaire that uses a 7-point verbal descriptor scale to assess discomfort due to gastrointestinal symptoms, with responses ranging from 1 (none) to 7 (very severe). The items can be grouped into 5 domains: abdominal pain (3 items), reflux syndrome (2 items), indigestion (4 items), diarrhea (3 items), and constipation (3 items). Figure 2 describes the administration schedule of questionnaires throughout the phase 2b study.

#### Dimensionality and scoring.

Interitem polychoric correlations were estimated for the items composing the mRESQ-eD. These correlations formed the basis for subsequent factor analysis and item-response theory (IRT) models.

Factor analysis was used to determine the way that items aggregate together to define relevant domains of interest for the mRESQ-eD. Oblique rotation of the ultimate factor solution was then performed to obtain the most interpretable structure.

To explore the presence of subdomain scores, 2 general IRT models, Samejima graded response model and a Rasch analog of the graded response model were used (17). In addition, a bifactor model was evaluated so that the total mRESQ-eD score could be calculated (18). IRT models were parameterized using the dimensionality structure as determined by the exploratory factor analyses.

After daily scoring was determined, summary scores over a patient's last week of daily administrations were determined to help summarize daily diary data.

#### Reliability.

Reliability was assessed in 2 ways: internal consistency and retest. Internal consistency was estimated based on the final modeling results that informed patients' scores (19). An estimate of ω ≥ 0.70 indicated acceptable internal consistency for the mRESQ-eD (20).

Retest reliability consisted of measuring the degree to which the mRESQ-eD yielded similar scale scores at different time points in symptomatically stable patients. This type of reliability was estimated using a 1-week retest interval that was obtained in the pretreatment period by correlating the first and second week mRESQ-eD scores. The absence of change in health status was operationalized by identifying patients for whom no change was observed in either weekly symptom relief or bothersomeness ratings. For these 2 groups of patients, repeated measures models using data from weeks-2 and -1 were fit for the mRESQ-eD scores from which estimates of retest reliability were based on the 2-way random intraclass correlation coefficient (ICC [2,1]) described by Shrout and Fleiss (21).

#### Validity

Two types of validity assessments were conducted. Construct validity was computed between the mRESQ-eD scores and the GSRS-Self scores at multiple follow-up assessments.

Convergence was estimated using polyserial correlations, given the ordinal response distribution for the symptom relief items and the continuous nature of the symptom scores with which the items were correlated. As is customary for these types of analyses, the prespecified criterion for acceptable validity was a correlation of ρ ≥ 0.40.

Known-groups validity was estimated by computing weekly-average mRESQ-eD scores and comparing the magnitude and significance of differences across levels of symptom bothersomeness for pretreatment and symptom relief for post-treatment. Mixed effects models were used to test these effects and compute corresponding effect sizes, measured by the semipartial η^2^ (22). Predictors for these models were relief, as measured by the symptom relief items and study week.

#### Responsiveness.

To assess responsiveness of scores, symptom relief ratings were used as anchors. The definitions of Clinically Important Improvement (CII) and Minimal CII (MCII) were defined as patients who reported “Moderate” symptom relief and “Somewhat” symptom relief at week 8, respectively. Percent changes from baseline, defined as week 1, were computed for each mRESQ-eD score, and empirical cumulative distribution functions were plotted. The median of the CII and MCII groups were taken to represent important improvements for determining a suitable “responder” criterion to aid in the interpretation of change scores.

## RESULTS

### Content validity study

#### Study population.

The sample of cognitive interview patients represented a range of ages and races. Gender was almost equally represented with a slight skew toward women (56.7%). Complete demographic information of the cognitive interview sample is provided in Table 1.

**Table 1.**
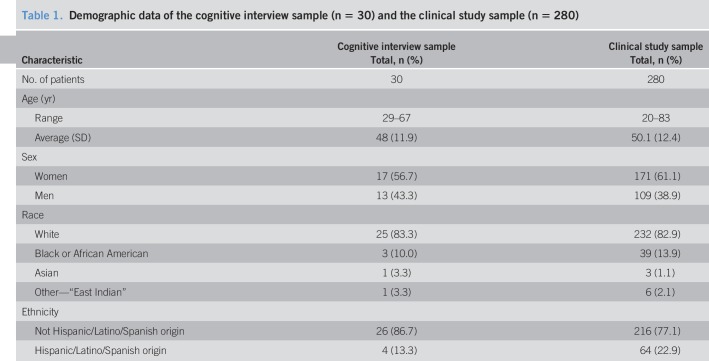
Demographic data of the cognitive interview sample (n = 30) and the clinical study sample (n = 280)

#### Cognitive interviews.

The draft mRESQ-eD contained 10 items relevant to heartburn, regurgitation, and esophageal signs and symptoms of GERD. Overall, the items, instructions, and response options of the mRESQ-eD were well understood and clear across most of the sample. Six patients encountered some level of difficulty with the terminology in the questionnaire, including the terms “hoarseness” and “regurgitation.”

Based on the results of the cognitive interviews, minor semantic changes were made to improve item clarity, but all item concepts were retained from the original 10-item mRESQ-eD as developed. Furthermore, the mRESQ-eD instructions were modified to improve consistency across the instructions and items. Namely, the recall period in the instructions was revised from “since waking today” to “over the past 24 hours” to match the recall period used in the items (i.e., “Over the past 24 hours”).

### Psychometric evaluation of the mRESQ-eD

#### Demographics for the psychometric validation.

The clinical study sample skewed women (61.1%) and represented a range of ages and races. Complete demographic information of the modified intent to treat clinical study sample is provided in Table 1. Of the 280 patients randomized into the clinical study, 9 patients received an old diary version and were thus removed from the validation analyses (Figure 1). Thus, 271 patients' data were included for validation. Among the 271 patients included, 141 (52.0%) had erosive esophagitis.

#### Dimensionality and scoring.

Results from the psychometric modeling supported the elimination of item 10 (cough frequency) from the 10-item mRESQ-eD because the item was determined to be redundant with item 6 (cough severity). Models were generated without each item (i.e., item 6 [cough severity] and item 10 [cough frequency]) to determine which item could be deleted with the least impact on model fit; results indicated that item 6 provided a better overall fit and was thus retained. Items 1 (heartburn severity) and 2 (burning feeling behind the breastbone or in the center of the upper stomach severity) from the 10-item version were carefully evaluated and found to have equivalent contributions to the psychometric model. After careful assessment with FDA reviewers, it was decided to remove item 1 (heartburn severity) and retain item 2 (burning feeling behind the breastbone or in the center of the upper stomach severity), which subsequently became item 1 in the 8-item version of the mRESQ-eD.

Ultimately, the psychometric modeling of the 8-item mRESQ-eD supported 3 daily scores: heartburn, comprising the highest severity between item 1 (burning feeling behind the breastbone or in the center of the upper stomach severity) and item 2 (pain behind the breastbone or in the center of the upper stomach severity); regurgitation, which is the maximum of item 6 (how often did you experience regurgitation) and item 7 (how often did you experience an acid or bitter taste in the mouth); and the total GERD score based on all 8 items. Each daily score was then summarized over a given study week.

For certain psychometric analysis purposes, heartburn, regurgitation, and total GERD scores were determined on a daily basis. In accordance with the psychometric modeling, assessments of model-based scoring and simple sum scoring procedures produced highly correlated results at baseline (ρ > 0.98). Therefore, the decision was made to use classical test theory scoring rather than model-based methods.

Heartburn scores were computed based on items 1 and 2. For each day, the maximum rating (i.e., worst severity of symptom) between the items was taken as a daily score. Weekly means of the daily maximum were then computed for each patient.

Regurgitation weekly scores were computed similarly to the Heartburn weekly scores but instead based on items 6 and 7. Item 8 was found to be part of the regurgitation/reflux construct when subjected to factor analyses, but it was determined to be distal to the other 2 items regarding regurgitation from a conceptual perspective. Therefore, although retained in the mRESQ-eD item set and in the total GERD score, item 8 was not included in the daily or weekly regurgitation scoring algorithm.

These weekly scores were then assessed for psychometric properties, including reliability, validity, and responsiveness to change.

#### Reliability.

Model-based internal consistency for the mRESQ-eD was found to be high (ω = 0.95), easily meeting the usual threshold. The classical test theory equivalent for the sum scores supported these results with coefficient α = 0.90, which was also well above the usual threshold of 0.70.

The retest models showed good fit to the data. The ICC (2,1) as defined by Shrout and Fleiss (21) and effect size estimates were calculated for the time effect. Heartburn weekly scores showed a good ICC of 0.68 (95.0% confidence interval of [0.35, 0.90]) in the presence of a moderate effect size (as given by Cohen [22]) of η^2^ = 0.09. Regurgitation weekly scores resulted an ICC of 0.74 (95.0% confidence interval of [−0.31, >0.99]) with an effect size of η^2^ = 0.09. Modeling of the total GERD score showed very similar results (ICC = 0.69, 95.0% confidence interval of [0.38, 0.90], η^2^ = 0.10).

#### Validity.

**Figure 3.**
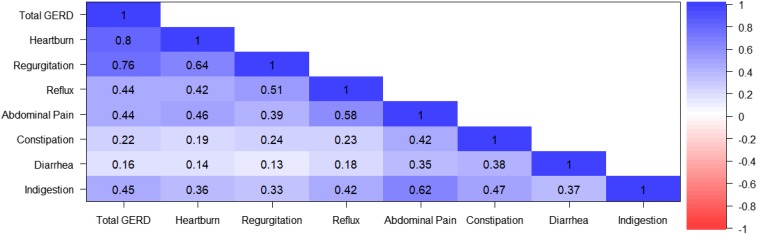
Heatmap of correlations between modified Reflux Symptom Questionnaire—electronic Diary scores and Gastrointestinal Symptom Rating Scale–Self-Administered Version scores at baseline. GERD, gastroesophageal reflux disease.

Weekly scores correlated positively with GSRS-Self items that contained concepts related to GERD (i.e., “Reflux,” “Abdominal Pain,” and “Indigestion”), with moderate correlations between 0.39 and 0.51 (Figure 3). Correspondingly, GSRS-Self items “Constipation” and “Diarrhea” correlated weakly with the mRESQ-eD, with score values between 0.13 and 0.24.

Known-groups validity were acceptable to the extent that health states were ordered with worse outcomes, demonstrating higher conditional means on the mRESQ-eD scores. In addition, paired comparisons that reflected significant differences and corresponding η^2^ ≥ 0.1 contributed to the evidence supporting the detection of known-groups validity. The mixed effects model results show significant effects of all model parameters. Effect sizes were extremely high for the known groups with η^2^ values, which was much larger than the usual threshold for a large effect size (i.e., η^2^ > 0.14). These results indicate that the heartburn and total GERD scores are quite effective at discriminating between the symptom relief groups.

#### Responsiveness.

**Figure 4.**
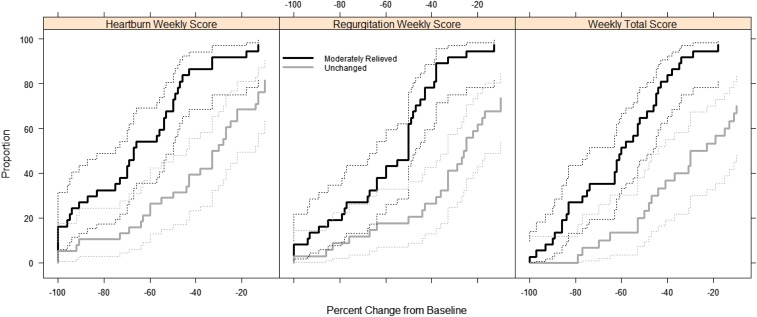
Empirical cumulative distribution functions of percent changes from baseline to study week 8 for the modified Reflux Symptom Questionnaire–electronic Diary scores with 95.0% confidence interval bands.

The heartburn score showed an MCII of 44.0% over 8 weeks, suggesting that the median response for patients indicating somewhat heartburn relief after 8 weeks was a 44.0% improvement over baseline (Figure 4).

In the case of the regurgitation score, the CII and MCII were found to be quite close in value, with patients indicating some form of relief by approximately 50.0% after 8 weeks of treatment.

The total GERD score showed an MCII of 50.5% over 8 weeks, demonstrating that the median response for patients indicating somewhat heartburn relief after 8 weeks was a 50.5% improvement over baseline.

### History of instrument modifications and final conceptual framework after psychometric evaluation

The RESQ-eD and subsequently the mRESQ-eD underwent several changes after concept elicitation and cognitive interviews as well as psychometric evaluation. A history of instrument modifications is summarized in Table 2.

**Table 2.**
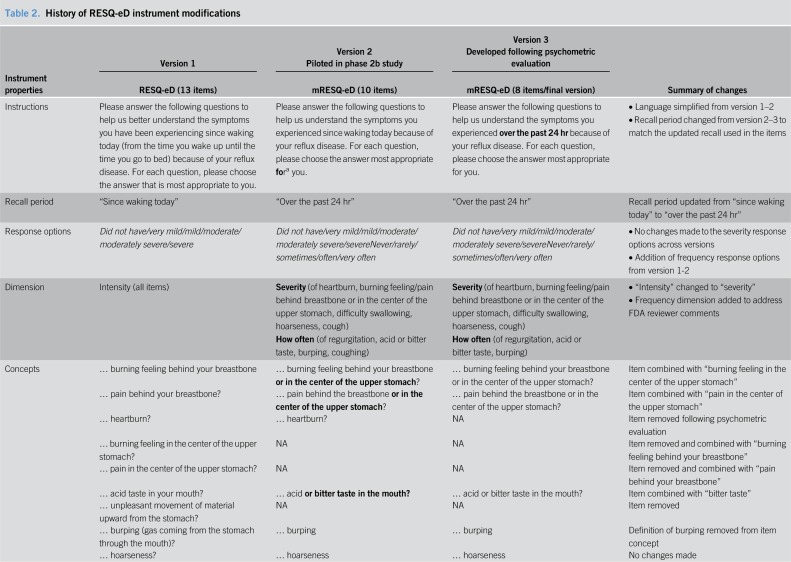

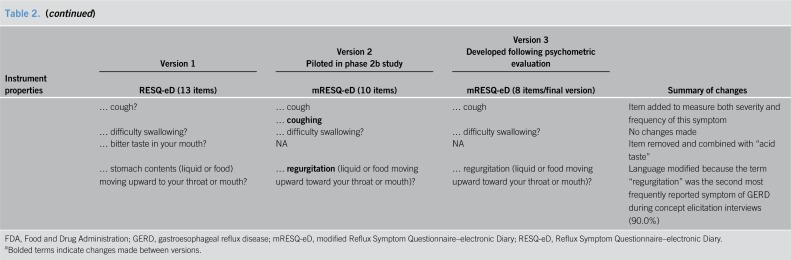
History of RESQ-eD instrument modifications

Once the final modification to the mRESQ-eD was made, the conceptual framework was revised and finalized. In the confirmed conceptual framework for the 8-item mRESQ-eD, 8 items form 3 symptom domains. The relationships are illustrated in Table 3.

**Table 3.**
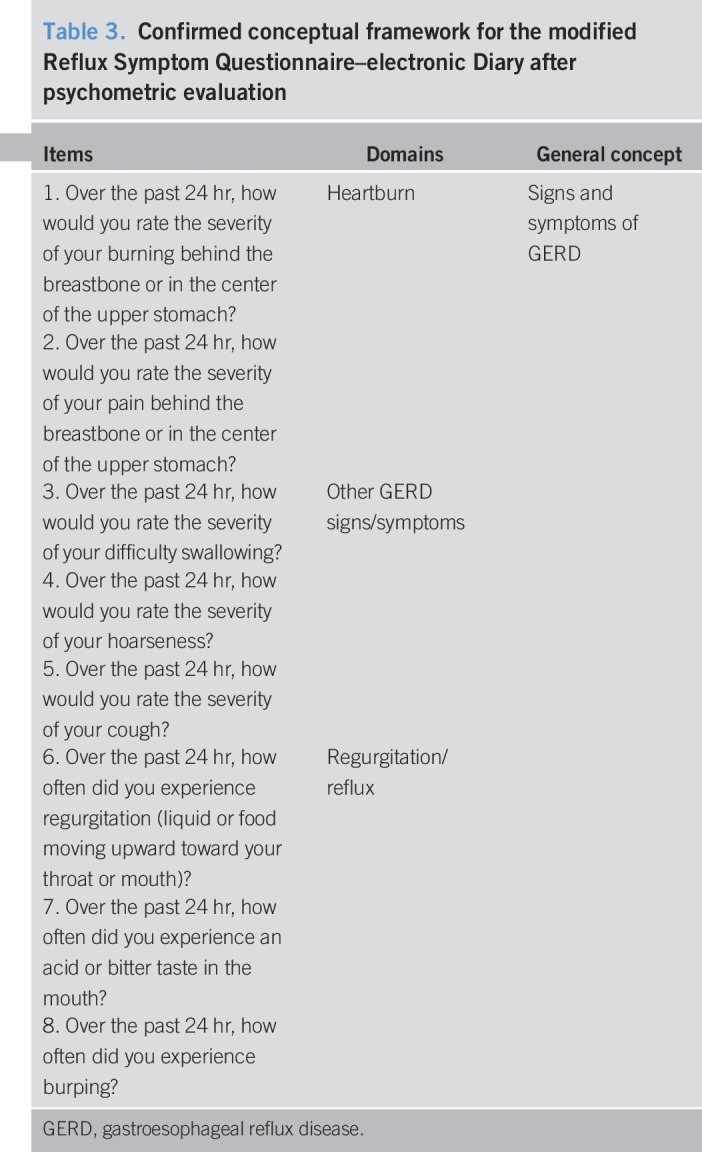
Confirmed conceptual framework for the modified Reflux Symptom Questionnaire–electronic Diary after psychometric evaluation

## DISCUSSION

When adapting a PRO instrument—vs developing one *de novo*—careful consideration should be given to the original instrument. Specifically, if there were errors made in the initial development process (e.g., missed concepts not included in the original instrument), they would carry over into the modification process because the instrument would be conceptually incomplete and subsequent efforts to modify the instrument would be unsuccessful. The extensive process undertaken during the modification of the original RESQ-eD likely mitigated any such errors because the additional qualitative work allowed further concept exploration to refine the instrument. The content validity of the original 13-item RESQ was established with patient input based on work conducted by the original developers (10). Subsequently, the content validity was re-established after additional concept elicitation interviews with patients enrolled in a phase 2a clinical trial for the treatment of persistent GERD. Results from these interviews led to the reduction of the 13-item RESQ-eD, which resulted in the 10-item mRESQ-eD. Content validity was further evaluated during cognitive debriefing interviews, and after these interviews, all 10 items were retained for psychometric evaluation. Subsequent to the psychometric evaluation, a total of 8 items were retained in the final version of the mRESQ-eD (as demonstrated in Table 3).

The adaption and modification of the RESQ benefitted from the existing body of work because this research was able to take advantage of the findings from previous research. One example was the focus on the heartburn domain during the psychometric evaluation of the mRESQ-eD based on findings from the initial development work and reaffirmed during cognitive interviews. The results from both activities highlighted that the concept of heartburn could be described in several ways, and particular attention was given to the necessity of having multiple items measure the same concept. Item 1 (heartburn severity) was removed from the final mRESQ-eD with the rationale that items 2 (burning feeling behind the breastbone or in the center of the upper stomach severity) and 3 (severity of your pain behind the breastbone or in the center of the upper stomach) of the 10-item mRESQ-eD represented elements of heartburn, and therefore, the original item 1 was not necessary.

Overall, reliability of the 8-item mRESQ-eD was very good and internal consistency was high, as measured within the psychometric modeling (ω = 0.95). Retest reliability was also acceptable with heartburn weekly scores showing good ICC with a moderate effect size. In addition, the regurgitation weekly score showed good ICC; however, the confidence interval associated with the regurgitation ICC was inconclusive because its lower bound was less than zero. This could be due to the variability within the sample. The patients in the current study were not specifically recruited for regurgitation, and therefore, regurgitation may be a more variable occurrence than a concept such as heartburn and may also be less salient than other aspects of GERD in this patient set. However, the moderate effect size associated with the regurgitation ICC indicated stability of the correlation. The moderate effect size could indicate a consistency for a subset of the patients in the current study, but that consistency is diluted across all patients. These seemingly incongruent results should be investigated in future research. The ICC associated with the total GERD score was also found to be good with a moderate effect size, indicating acceptable retest reliability.

In addition, validity measures for the 3 scores were found to be positive. Convergence was established between the mRESQ-eD scores and the GSRS-Self “Reflux,” “Abdominal Pain,” and “Indigestion” items. Although below the usually accepted criterion of 0.40, the correlation between regurgitation and abdominal pain (ρ = 0.39) still indicates that the 2 variables share 15.0% of their common variance (0.39^2^ = 0.152), which is a small to medium effect size; furthermore, this effect size is not insignificant, given the constructs relating these scores. The so-called known-groups showed excellent results with large effect sizes.

The modification and validation of the mRESQ-eD follows guidelines established by FDA to continuously adapt instruments to support PRO qualification (23). The instrument was identified for the appropriate context of use in patients with persistent GERD, significant qualitative work was conducted to modify the instrument and establish content validity based on patient input, and cross-sectional and longitudinal evaluation of measurement properties were explored to provide rationale to further modify the instrument and establish the final 8-item version. This research demonstrates that *de novo* PRO development is not the only path for creating content valid and psychometrically sound instruments; with supportive qualitative and quantitative work, an existing instrument can be refined to further confirm the appropriateness of the instrument for its intended use.

The mRESQ-eD is a reliable and valid PRO instrument with good psychometric properties that may be used to characterize the cardinal symptoms of persistent GERD. The mRESQ-eD may be considered for inclusion in clinical trials for persistent GERD and potentially positioned, in consultation with FDA, as primary or secondary endpoints to characterize treatment benefit.

## CONFLICTS OF INTEREST

**Guarantor of the article:** Jennifer Hanlon, MPH.

**Specific author contributions:** J.H. and D.R.: study concept and design, and manuscript review. D.A.A.: psychometric concept and design, data management, analysis, and manuscript writing and revisions. B.W.: study concept and design, review and guidance of data analysis process, and manuscript outline development. M.L.C.: study design, data analysis, and manuscript writing. K.S.: data collection, analysis, and manuscript writing and revisions. C.G.: data collection, analysis, and manuscript writing and revisions.

**Financial support:** This study was funded by the Ironwood Pharmaceuticals.

**Potential competing interests:** J.H., D.R., and J.Z.S. are or were employees of Ironwood Pharmaceuticals at the time this research was conducted. Endpoint Outcomes received consultancy fees from Ironwood Pharmaceuticals. D.A.A., B.W., M.L.C., K.S., and C.G. are employees of Endpoint Outcomes.

**Study Highlights**

**WHAT IS KNOWN**

✓ GERD is a common, chronic gastrointestinal illness.
✓ Instances of persistent GERD represent a sizeable portion of all patients with GERD.
✓ The RESQ-eD was developed to assess signs/symptoms of persistent GERD.

**WHAT IS NEW HERE**

✓ The RESQ-eD was modified based on patient input to improve the content validity of the measure.
✓ The mRESQ-eD demonstrates acceptable and valid psychometric properties.

**TRANSLATIONAL IMPACT**

✓ The mRESQ-eD may be considered for inclusion in clinical trials for persistent GERD to characterize treatment benefit.
✓ *De novo* PRO development is not the only path for creating content valid and psychometrically sound instruments.
